# Something New: An Interview with Radoje Drmanac

**DOI:** 10.1371/journal.pgen.1001049

**Published:** 2010-08-19

**Authors:** Jane Gitschier

**Affiliations:** Department of Medicine and Pediatrics, University of California San Francisco, San Francisco, California, United States of America

Each year when I attend the American Society of Human Genetics meeting, I stroll up and down the vendor exhibits to see what's new, and, at the most recent meeting, I noticed one booth that stopped me in my tracks. The company was Complete Genomics, and I asked the rep about the cost of sequencing small stretches of the genome for a project in the lab. She told me they only do “complete” sequencing—full human genome sequencing. “That's why it's called ‘Complete’,” she added. OK, I get it—so how much does it cost? “$20,000 per sample if you order sequence for eight samples.”

Figuring that anybody who can produce accurate full sequence for that kind of money has to have a clever idea, I looked at the exhibit materials more closely. Part of their success comes from employing “rolling circle” replication to amplify a small circular piece of genomic DNA that is interrupted by four adapters. The long, single-stranded DNA that spools off collapses in on itself by base-pairing of separated, inverted repeats lying in the adapters, as I later learned, forming a tight and highly charged “DNA nanoball.” These nanoballs can be uniformly distributed onto a prepared grid, and the high concentration of target sequence allows for very quick and cost-effective imaging. The four adapter sequences then serve as the anchors for sequencing—not by enzymatic extension, but rather by sequential hybridization and ligation of pairs of oligonucleotides for each base position.

**Image 1:**
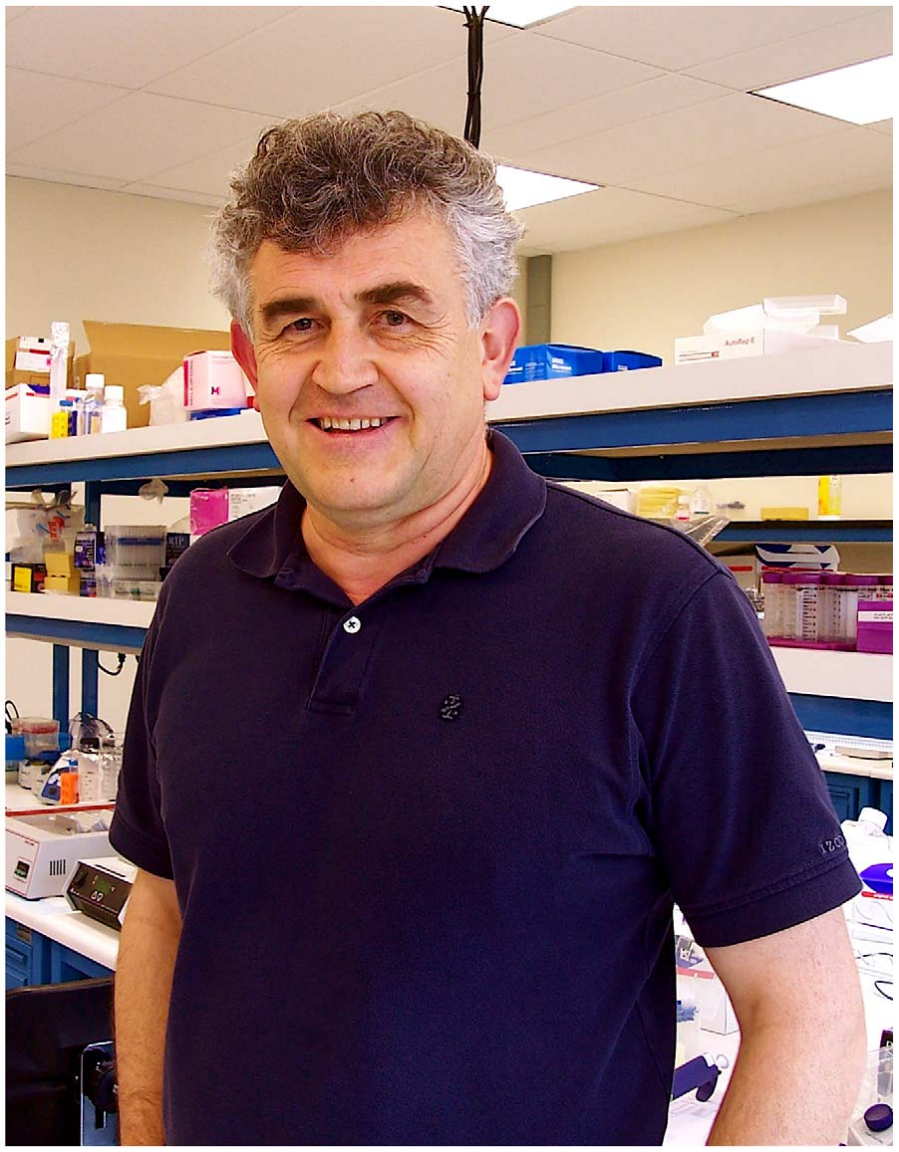
Radoje Drmanac.

The exhibit drew out my inner geek, and I wanted to learn more. The rep pointed me in the direction of the inventor and company co-founder, Radoje Drmanac (Image 1), informally called “Rade,” who was deep in conversation with someone else, so I moved on. A few months later, I was prompted by his first-author publication in *Science* reporting a US$4,000 genome to delve a little deeper. I discovered that Rade's scientific career has been driven by his ambition to sequence human genomic DNA using oligonucleotides. In 1988, while still a graduate student in his native Serbia (Yugoslavia at the time), he published his first idea, dubbed “sequencing by hybridization” (SBH), in *Genomics* and followed that up with a publication in 1990 about doing PCR in emulsion to produce abundant template. After working at Argonne Laboratories and forming two prior companies, he co-founded Complete Genomics, finally bringing to fruition his goal for efficient sequencing of the human genome.

I got the feeling that Rade had the vision, tenacity, and spirit of invention that would make for a good interview. In late March, I drove through what I hoped were the final rains of the Bay Area wet season (they weren't!), to meet him at Complete Genomics in Mountain View, just past the colorful Google campus.

**Drmanac:** You probably want to know how to pronounce my name.

**Gitschier:** Yes!

**Drmanac:** I can *misspell* it for you, so you can pronounce it. It's like D-E-R-M-A-N-A-T-S. Two mutations.

**Gitschier:** Normally when I interview someone, I like to start with a little biographical background and work my way up, but in this case, I want to do the opposite. Let's start with where we are now, and in the course of that, we'll get some background. And then we'll go back a little deeper.

**Drmanac:** That's much better. I'm old enough that you'll never come to the present if you start from Yugoslavia!

**Gitschier:** Well, you're younger than I am! Let's start by educating me about the technology that Complete Genomics is using; I want to make sure I understand it.

First, making the template for the nanoballs—you start with fragmented human genomic DNA, then add some half adapters that ligate to the ends of the fragments and to each other [forming a closed circle with the fragment ends—the “mate pairs”—separated by “adapter 1”]. Then, you add a Type II restriction enzyme that recognizes a sequence in the adapter and cuts within the genomic insert somewhere nearby.

**Drmanac:** Yes, the first one we used cuts about 13 bases away. But there are others that cut 25 bases away.

**Gitschier:** It must cut in only one direction.

**Drmanac:** We have [recognition] sites [in adapter 1] for it to cut in both directions, but one site is methylated and the insert is methylated, so only one site can be recognized. After we make the first cut and stick in the second adapter [adapter 2], we use a few cycles of PCR to remove the methylation.

**Gitschier:** Cool.

**Drmanac:** Just DNA engineering. Then, we methylate everything again except this site [the other site in adapter 1] because we keep this single stranded. Then, when you close the circle, you form the new unmethylated site, going in the opposite direction.

**Gitschier:** And I take it that it's the same restriction recognition site, so you make another cut in the opposite direction 13 bases away from the first adapter.

**Drmanac:** Correct. There aren't that many good restriction enzymes that do this, so we use the same one. So that is adapter 3 now [that is inserted]. And adapter 4 is quite easy. A recognition site is present in both adapter 2 and 3 and [the enzyme] cuts 25 bases away from each, and we remove the 400 bases that we don't need and replace that with adapter 4. Quite simple on paper! But it took several exceptionally talented scientists a few years to implement.

**Gitschier:** OK, so now you have a small circle of human genomic DNA with four adapters inserted. Adapter 1 separates the two mate-pair sequences, adapter 4 replaces most of the genomic DNA in between, and adapters 2 and 3 provide additional “anchor” sites in the mate-pair sequences to allow you to extract more sequence information. And you figure out what these bases are by oligonucleotide hybridization.

But before we talk hybridization, we need to talk about how this template is amplified into DNA nanoballs—I love these things.

**Drmanac:** Yeah—it's the old famous Φ29 enzyme from 20 years ago. In 1989, Blanco et al. showed they could amplify M13 plasmids. It has very strong displacement activity, and it is highly processive. It goes up to 100 kb without stopping. So that was all known.

You know, my whole life I've just used what is available—in new combinations and with some modifications—to change the meaning or purpose of existing discoveries. So this was one example. You get this concatemer of 250 bases and you can make 500 copies.

**Gitschier:** So this long concatemer allows you to have this very strong signal down the road.

**Drmanac:** Correct. Everybody is now trying to do single molecule sequencing, but what we are doing is as simple as you can get and you don't have to worry about single molecule detection, which is costly and error-prone. We are trying to avoid those two problems. I think we are right, at least for now, because the limiting factor is the imaging step.

**Gitschier:** So you can do your imaging faster.

**Drmanac:** Yeah, short exposures. We can use the nanoballs to make a perfect grid whereas everybody else is using random positions [of the template DNA]. The perfect grid allows us to do the most efficient imaging. You can align the CCD pixels to the nanoballs. We are going down to one pixel per nanoball; today we use two pixels per nanoball to be cautious.

**Gitschier:** And that's because before you put the nanoballs on there, you set up a grid so that each…

**Drmanac:** …active site matches the size of the nanoball. When it is compact, it's about 200 nM. It has a strong negative charge, because it's a small particle with ∼100,000 negative phosphate groups, so the nanoballs repel each other. So in solution, there is no entanglement; they are forcing each other out to be in perfect separation.

**Gitschier:** So how does the nanoball form in the first place with so much negative charge—why don't they stretch out?

**Drmanac:** We put palindromes of, say, 7 As and 7 Ts—not adjacent—into the adapters. When it starts out, it is single-stranded DNA and hybridizes to itself. Since it's single-stranded, it's flexible and easy to make into a ball. And we intentionally made its stability borderline so the palindromes would pair on and off—the first binding with the second initially, but eventually the first binds with the tenth copy, so it forms a 3-D structure.

**Gitschier:** And you can regulate that by the temperature or…

**Drmanac:** …with the size of the palindrome and the reaction conditions. It's really nanoengineering. Very simple, but it works.

The other interesting thing about this circle: it has more synthetic DNA [with the four adapters] than genomic DNA. So there is no GC bias between nanoballs because most of the DNA is shared. That's why making the nanoballs is so reproducible and the size is so consistent. And if you look at the surface, there is 10 times as much DNA per unit surface area than you can get with a bridge PCR amplification.

**Gitschier:** And it's already single-stranded, so you're all ready to go for the hybridization.

**Drmanac:** It's pretty simple with almost no disadvantages.

**Gitschier:** How was the idea for a DNA nanoball generated?

**Drmanac:** Matt Callow, a scientist from my group, thought of it. We got a Biodefense grant to develop this genomic array-based sequencing using ligase, and we tried all kinds of ways to do amplification. Matt came one day and said, “We can use this old concept of rolling circle replication.” Ah, this is great because you don't need oil [for an emulsion PCR step]. In situ amplification on a surface is always problematic. I expanded on that idea to add the palindromes, and we played with that for a long time, so that the amplified DNA will create a particle in solution. We can match the size of the particle with the size of the spots on the array to assure a single DNA particle per spot. Patterned DNA nanoarrays are critical for our success.

**Gitschier:** What about using the type II enzyme and the adaptors?

**Drmanac:** I realized that with mate pairs, you don't need much of the sequence—even 20 bases is enough. But ligase doesn't allow you more than about five, so having extra adapters, which are inserted through the use of type II enzymes, provides us with extra stepping stones for ligase. And this will provide us with enough sequence data to analyze a complete human genome.

There is a paper that George Church published in *Science* in 2005, where he used a similar process, without the inserted adapters. And he read 13 bases on each mate pair, and that was good for sequencing *E. coli*, but it was not good enough for humans. So that was a simple, but critical element.

**Gitschier:** Now we're going to get to the sequencing by [oligonucleotide] hybridization.

**Drmanac:** That's quite simple.

**Gitschier:** Well, I understand it a bit, but not completely. You read two sets of genomic sequence adjacent to adapter 1 that are each 13–15 bases in length and two sets of sequence adjacent to adapter 4 that are each 25 bases in length.

**Drmanac:** Correct. That is for now; we will go up to all being 25 bases in length. But let's start with the first five bases. There is an anchor oligo that matches the end of the adapter. Then the sequencing oligo, which is always nine bases. Only one of the bases is informative, everything else is degenerate, and we read only five bases in each of them. You have a pool of probes, with one base being queried and the other eight positions being degenerate, meaning you have 65,000 [4^8^] different sequences for each of the four bases at each position.

We pipette this mixture over the array and then we ligate. Anything that can be ligated will ligate. The first five bases are all that matter—for ligase and for us—because we never read beyond the fifth base. We put the informative bases only in the first five positions, because ligase has good proofreading up to five bases. We make them nine bases total in length only for the footprint of the ligase.

**Gitschier:** But I don't understand how you keep track of which oligo is which.

**Drmanac:** It's all about this one informative base. If the DNA has C at this position, the probe with a G there will ligate, and it is labeled with a specific dye.

**Gitschier:** Well, I understand that, but there are going to be a lot of molecules in the collection that have a G there—in fact ¼ of them, and how do you know *which* one…

**Drmanac:** It doesn't matter! Because we don't read anything else.

**Gitschier:** OK, I get it now!

You are querying only one base at a time, not the whole stretch of five bases. You do repeated rounds of querying. So the first round you have a pool of oligos, where everything in the first position with a G, say, is tagged with one dye, and A another dye, etc. Then you must wash everything off and you go through with a new set of 256,000 probes, 65,000 with a G in the second position, etc., and you do that hybridization and ligation, wash that off, etc.

**Drmanac:** Exactly. And the probes we use are universal—one pool of probes for each of the five positions, and we never change that, so it is simple.

**Gitschier:** So, you wash off the ligase and remove the excess of the probes, and take an image.

**Drmanac:** Correct. After we have the image, we strip off the whole complex, getting back to the clean DNA for position 2. We use the same anchor oligonucleotide again and the next pool.

**Gitschier:** So this really is a bit different from the original concept you had in 1988 for sequencing by hybridization, where you had a stretch of DNA and you add a pool of labeled oligos to interrogate a complete six-base sequence, for example.

**Drmanac:** Yes, for all 4,096 possible hexamers, for example, I have to test 4,096 probes—if I have four colors, I can get down to about 1,000 tests, or 1,000 cycles.

Actually there was a paper published in *Nature Biotechnology*—I was so happy to see it—in 2008, and I cited it. They did exactly that. They used five-base probes and did 1,000 cycles and read the bacterial genome with 1,000 cycles.

**Gitschier:** OK, let's talk about how Complete Genomics got started. You had been working at Callida, a spin-off from Hyseq.

**Drmanac:** First, I met John Curson, our CFO and one of the three co-founders, whose son worked at Callida. John introduced me to Cliff Reid, our CEO. Cliff was interested in starting up a biotech company, so he called me. After founding multiple enterprise software startups, he decided to go into biotech. He went back to MIT to learn molecular biology. We licensed the IP [intellectual property] from Callida for genome sequencing—this is a typical VC [venture capital] approach. Companies have to be focused; otherwise they won't succeed. We decided to license the technology specific for that field.

In 2006, we got the first round of funding for $6 million. [That was] the birth of Complete Genomics. Twenty years of experience in these different technologies, and also IP, were very important to shorten our development time. That was a surprise, actually. Cliff was so successful in getting funding that we didn't need public awareness. We were in stealth mode for about three years. We had already sequenced the first human genome before we announced our existence.

**Gitschier:** What genome was it?

**Drmanac:** It was one of the HapMap cell lines. We decided not to go with a famous person. We just chose scientifically.

We were so focused. And one important thing: we decided we were not going to sequence a bacterial genome as a demonstration, not to focus on a thousand times smaller scale. We knew the process worked. It's not a new chemistry or new physics—it's all about scale. We decided to wait and sequence our first human genome. And I think we saved a year in development because we focused on that scale.

**Gitschier:** Why did you develop the idea that it would always be a “complete” sequence?

**Drmanac:** That was the sole focus from day one because that was what was missing. You can use existing technologies to sequence more species, but there was no way to sequence hundreds of thousands of human genomes.

If you are developing technology that is universal, you always compromise. Flexibility would be more important than capacity and cost. But if you focus on only the human genome, then you focus on scale and cost rather than flexibility. And that paid off. For example, our production instrument can sequence 18 genomes in one run. And a run is about 11 days. Two terabases per run.

It's a big project. Even today we're not doing the scale that we need to do to understand a disease. If we take one type of tumor—100 pairs of sequences from the same type of tumor [tumor and normal tissue from each patient]—then we will know real pathways. We will have soon a paper with Genentech; in one pair you see 50,000 mutations. So to figure something out, you have to do 100 pairs. We want to do 1 million genomes for research.

**Gitschier:** How many have you done so far?

**Drmanac:** Last year, we delivered 50 to our partners. We just published a paper with Lee Hood—two parents and two children with Miller syndrome and a lung disease similar to CF [cystic fibrosis]. And they found genes causing both diseases—compound heterozygotes in both cases.

Of course, having the parents helps you confirm the findings, since there are new mutations and sequencing errors. Our accuracy is one of the best in the industry, but still, there are tens of thousands of errors, because our genome is so big. One error in 200,000, but there are 3 billion bases. So we can remove 75% of errors by comparing parents and kids. They took the 20,000 errors they thought they had and tested them and found that 100 were actually not sequencing errors, but de novo mutations.

**Gitschier:** OK. Let's finish up with the beginning. You were born in Serbia, which was then part of Yugoslavia.

**Drmanac:** Yes. Actually I was born in 1957, on December 25th, in a little village, but my grandma hid me until 1958. She didn't want me to be the youngest in school and in the army, because she thought it would be a disadvantage [because you are enrolled and enlisted based on the calendar year of your birth], so she made my official birth date January 2nd, 1958. That's what is on my birth certificate. It was a little village, so it was possible to do this.

Having a grandma around is great for kids. I had a nice long childhood in my village, always playing outside or helping with farm work. We were producing almost all our food; we were buying just a few things. We had to do many different things successfully; otherwise, we would not have enough food to eat.

When it became time to go to school, my father was working in the city, about four miles away, and I went to school in the city with him to get a better education. I walked down the hill and up the hill every school day for 12 years.

My great-grandma never had an education, she couldn't read or write, but she was very wise. She was consulted by many people in the village about the calendar or the moon, and somehow she knew everything. She influenced me that you don't go to school so that you can get work—anybody can get work. You go to school to *discover* something or to *invent something new*!

**Gitschier:** Ah, it all started with that very wise old lady! Tell me when the idea of sequencing by hybridization took hold?

**Drmanac:** I was a graduate student at the University of Belgrade and Kary Mullis had just invented PCR. I think this discovery is so important they should give him two Nobel prizes! I was telling a new member of our team about PCR and how we could use it for whole-genome mapping. And at that moment I realized that if we had all possible primers of a given length, we could do sequencing. I immediately became obsessed with this idea of sequencing the entire genome at once without using gel sequencing. I couldn't think about anything else. I would take my children to the park—I have twins, a boy and a girl—and so many times they would deliberately ask me a question to which I was supposed to answer “no” and I would automatically say “yes.” And they would say, “Dad, you are not listening to us, you were supposed to answer “no” on this question.” I am so thankful that my wife was able to cope with me. She turned from a medical doctor into a molecular biologist, on her own insistence, and we have worked together ever since.

I'd like to tell you this. The SBH paper got rejected by *PNAS*, *Nature*, *NAR*, and maybe a few other journals. Theories of biochemical methods were not valued at that time. But my career depended on it. Finally, thanks to Victor McKusick, it got published in *Genomics*. He recognized the value of large-scale human genome sequencing. I met him several years later and thanked him for saving my career.

**Gitschier:** Then for a post-doc you moved to Hans Lehrach's lab in London, again working on oligo hybridization. Was it a bit of a culture shock to move to London?

**Drmanac:** That is interesting. You know, Yugoslavia was a communist country, and people think we were very limited. But that wasn't true. We had a lot of influence from the West: foreign aid and investment to prevent Russian expansion in Yugoslavia and good lab equipment. In 1988, we got a US Department of Energy genome program grant: 150,000 real green US dollars to work on SBH. Quite interesting—a US government department that develops atomic bombs was funding biotech research in a communist country.

There were many cultural opportunities, education was good, and people had jobs. The only off-limits thing was politics. We did not have a multiparty system. If you disagreed in politics, you could end up in jail or get shot. But other than that, it was really good. I didn't care about politics.

So when I went to London, I saw that we had better equipment and laboratories in Belgrade than in London. And I had majored in Molecular Biology—they didn't have such a major in England. They were bound by a long tradition, but we in Belgrade weren't. We had the opportunity to do something new.

